# Reporter-Based Isolation of Developmental Myogenic Progenitors

**DOI:** 10.3389/fphys.2018.00352

**Published:** 2018-04-05

**Authors:** Eyemen Kheir, Gabriella Cusella, Graziella Messina, Giulio Cossu, Stefano Biressi

**Affiliations:** ^1^Centre for Integrative Biology (CIBIO), University of Trento, Trento, Italy; ^2^Dulbecco Telethon Institute, University of Trento, Trento, Italy; ^3^Human Anatomy Unit, Department of Public Health, Experimental Medicine and Forensic, University of Pavia, Pavia, Italy; ^4^Center for Health Technologies, University of Pavia, Pavia, Italy; ^5^Department of Biosciences, University of Milan, Milan, Italy; ^6^Division of Cell Matrix Biology and Regenerative Medicine, Manchester Academic Health Science Centre, University of Manchester, Manchester, United Kingdom

**Keywords:** embryonic myoblasts, fetal myoblats, FACS, reporter lines, myf5

## Abstract

The formation and activity of mammalian tissues entail finely regulated processes, involving the concerted organization and interaction of multiple cell types. In recent years the prospective isolation of distinct progenitor and stem cell populations has become a powerful tool in the hands of developmental biologists and has rendered the investigation of their intrinsic properties possible. In this protocol, we describe how to purify progenitors with different lineage history and degree of differentiation from embryonic and fetal skeletal muscle by fluorescence-activated cell sorting (FACS). The approach takes advantage of a panel of murine strains expressing fluorescent reporter genes specifically in the myogenic progenitors. We provide a detailed description of the dissection procedures and of the enzymatic dissociation required to maximize the yield of mononucleated cells for subsequent FACS-based purification. The procedure takes ~6–7 h to complete and allows for the isolation and the subsequent molecular and phenotypic characterization of developmental myogenic progenitors.

## Introduction

### Development of the protocol

**Figure 1:**
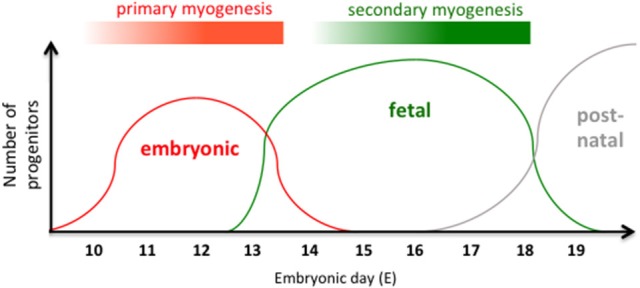
Intrinsically different myogenic lineages govern embryonic and fetal phases of muscle fiber formation. Schematic representation of the major phases of myogenesis that are occurring during skeletal muscle development. Embryonic progenitors that are responsible for primary myogenesis are predominating between E10 and E13, whereas fetal progenitors that are responsible for the secondary myogenesis are predominating between E14 and E18. Post-natal progenitors/stem cells are responsible for muscle growth after birth. The developmental time corresponding to the different phases of myogenesis in the mouse is indicated on the x-axis. Embryonic days are counted, considering E0.5 the morning of the vaginal plug. Myotomal cells giving rise to the early embryonic myotome is omitted from this scheme.

**Figure 2:**
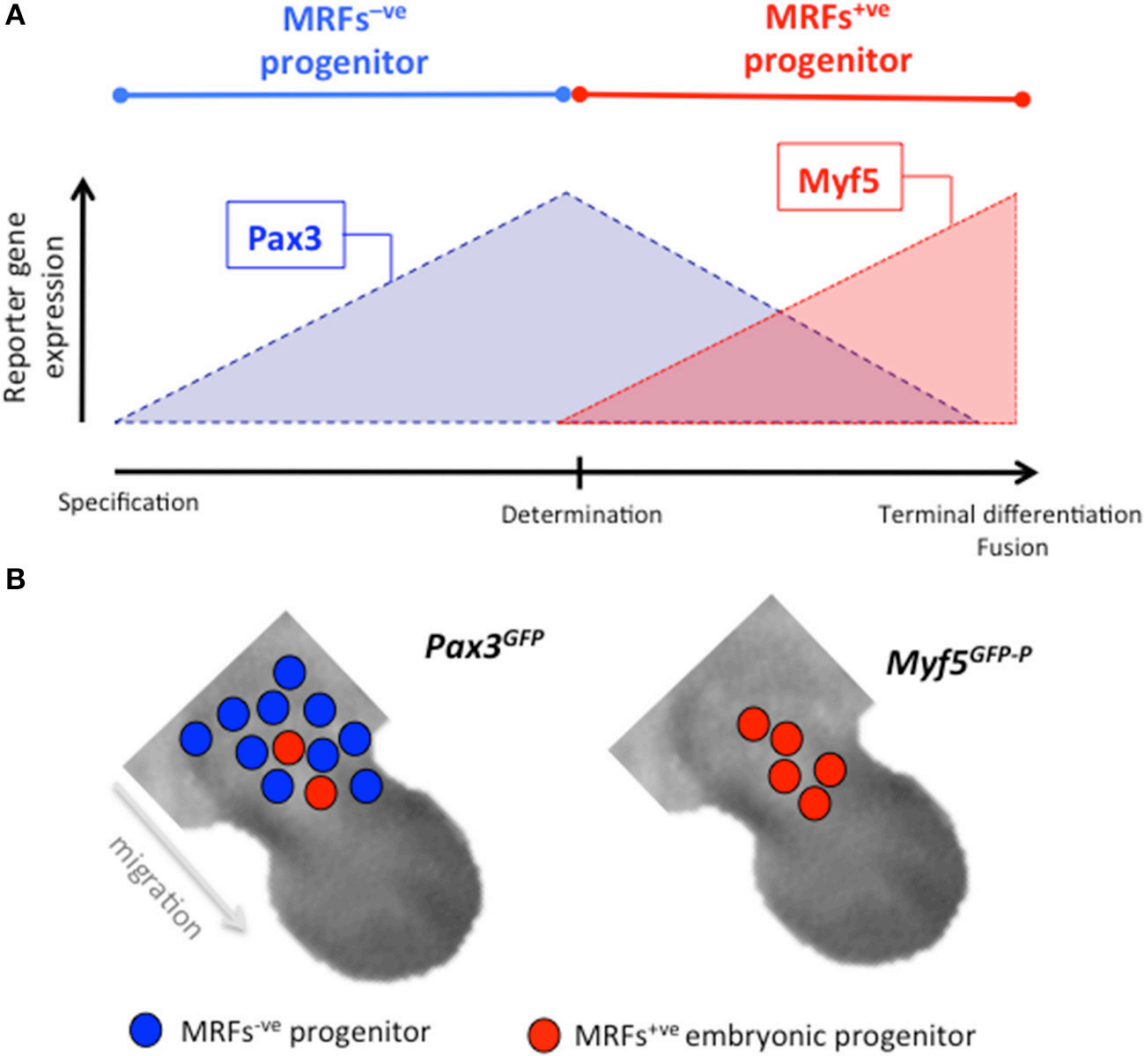
Discrimination between progenitors with different degree of differentiation. **(A)** Schematic representation showing the expression of the reporter GFP in *Pax3*^*GFP*^ and *Myf5*^*GFP*−*P*^ mice during the myogenic lineage progression at the embryonic stage. Different steps of the lineage progression leading to terminal differentiation are indicated on the x-axis. **(B)** Representation of the numerical proportion and position of MRFs^−ve^ and MRFs^+ve^ progenitors expressing GFP in limbs of *Pax3*^*GFP*^ and *Myf5*^*GFP*−*P*^ at E11.5. Note that only in the *Pax3*^*GFP*^ embryos GFP is expressed in migrating MRFs^−ve^ progenitors.

Skeletal muscle development consists in the formation of contractile muscle fibers by the progressive differentiation of distinct classes of progenitors that appear at subsequent developmental stages. It is among the most studied developmental processes (Figure 1) (Stockdale, 1992; Tajbakhsh, 2005; Buckingham, 2006; Biressi et al., 2007a; Murphy and Kardon, 2011). The first multinucleated fibers are formed approximately between E10.5 and E12.5 in the mouse, in a phase, which is referred to as primary myogenesis. During this phase, embryonic progenitors of somitic origin progressively commit to the myogenic fate and, upon migration to their appropriate muscle anlagen, terminally differentiate by fusing with each other into fibers that are called primary fibers. This lineage progression is a finely tuned process that depends on signals coming from the adjacent developing tissues and neighboring cells present in the sites of muscle formation or co-migrating with the myogenic progenitors (i.e. endothelial cells). During this process, a number of muscle-characteristic genes are serially induced in the myogenic progenitors as they progress from their initial specification to terminal differentiation. These genes include the members of the Pax family of specification genes Pax3 and Pax7 and muscle determination genes, such as the myogenic regulatory factors (MRFs) Myf5 or MyoD (Figure 2A). Only a fraction of myogenic progenitors terminally differentiates during primary myogenesis. The remaining are kept in a committed but undifferentiated state (likely depending on Pax7 expression) until subsequent fetal and post-natal phases of myogenesis. The fetal phase of muscle formation usually referred to as secondary myogenesis, peaks between E14.5 and E17.5 in the mouse. This phase depends on the fusion of MRF^+ve^ fetal myoblasts, in a process that is similar to that described for embryonic progenitors during primary myogenesis, although some of the fetal cells also fuse with primary embryonic myofibers. Despite some general similarities, studies from different laboratories, including our own, identified specific properties of embryonic and fetal myogenic progenitors, which characterize them as intrinsically different classes of cells terminally differentiating in muscle fibers with distinct biochemical and metabolic characteristics (Stockdale, 1992; Biressi et al., 2007a; Murphy and Kardon, 2011). In general, fibers formed during mammalian primary myogenesis (and myotubes formed from the fusion of embryonic progenitors *in vitro*) are predominantly programmed for a slow phenotype, whereas those formed during secondary myogenesis (and myotubes formed by fetal myoblasts *in vitro*) acquire a fast phenotype (Zhang et al., 1998; Wigmore and Evans, 2002). Although the activity of the nerves plays a well-documented role in fiber type specification, the phenotypic diversification of developmental myogenic progenitors during differentiation *in vitro* is occurring independently on innervation (Biressi et al., 2007b). This body of evidence highlights a distinct, intrinsically codified physiological function for muscle fibers formed at different developmental stages.

Myogenic progenitors represent only a minority of the cells that comprise the embryonic and fetal mesoderm. The heterogeneity of the cell types coexisting in the growing muscle, and the complexity of their interrelationships severely limit the type of questions about developmental mechanisms that can be reliably answered in the intact organism or in cells isolated from total mesoderm. Signals coming from the surrounding non-myogenic cells dramatically influence the characteristics and fate of muscle progenitor cells, complicating the interpretation of many previous observations.

Here we describe a protocol that allows the isolation of pure populations of myogenic progenitors at different developmental stages. We used this method to prove that embryonic and fetal myoblast are intrinsically different progenitors (Biressi et al., 2007b, 2008; Messina et al., 2010; Taglietti et al., 2016). We also used this protocol to separate embryonic progenitors with different degree of differentiation (Biressi et al., 2008; Boutet et al., 2010), and to define the developmental origin of the adult muscle stem cells (Biressi et al., 2013). The use of this procedure should further expand the opportunities to (1) investigate common and unique properties of specific subpopulations, (2) allow *in vitro* studies of molecules that regulate muscle cell growth and differentiation, (3) perform molecular and functional studies in the absence of confounding contaminants, (4) characterize cells at different stages of myogenic progression and differentiation, (5) investigate the intrinsic properties and phenotypic plasticity of muscle fiber subtypes in the absence of innervation, (6) isolate and analyze mutant myoblasts from genetically modified animals, and (7) use these cells in cell transplantation studies in animal models of human disease. The possibility of purifying specific cell subpopulations and analyzing them under controlled conditions offers the opportunity to investigate their intrinsic properties. Furthermore, the response of these cells to defined stimuli can conveniently be studied without the confounding presence of other cell types. With the advent of next-generation sequencing and single cell analysis, it becomes possible to dissect the molecular determinants governing cellular dynamics. Importantly, these techniques rely on the possibility of identifying and isolating specific cell populations. The definition of reliable procedures of cellular purification, such as those described in this protocol, offers the possibility to apply these investigative approaches to skeletal muscle development and differentiation.

### Comparison with other flow cytometry methods

Although several strategies have been proposed to isolate myogenic progenitors from adult muscles by flow cytometry (Gromova et al., 2015; Liu et al., 2015) until now only a few approaches for perspective isolation of myogenic cells from developing muscles have been reported. One method employs an enrichment step through Percoll density centrifugation followed by a subsequent selection using 90° light scattering (Yablonka-Reuveni, 1988). This method is reportedly effective in purifying myogenic progenitors from 10-day-old chicken embryos, but no evidence for its efficacy at later stages or in other organisms is documented. FACS-based approaches relying on antibodies recognizing specific markers have been conversely reported to purify fetal myogenic progenitors from both human and mouse muscles. Human fetal myoblasts are identified as melanoma cell adhesion molecule (MCAM)^+ve^ cells (Lapan and Gussoni, 2012), but if the same marker is effective in purifying myogenic progenitors from different species and stages is unknown. In the case of mouse fetal progenitors, the use of different combinations of markers that include integrin α7 (Itga7) have been proposed (Castiglioni et al., 2014; Tierney et al., 2016). Nevertheless, the preferential expression of Itga7 in fetal progenitors in comparison to their embryonic counterpart, suggests that this approach may not be extended to embryonic developmental stages (George-Weinstein et al., 1993; Biressi et al., 2007b). To date, the approach we describe here is the only one that has been proven to effectively purify cells from both embryonic and fetal stages.

The reporter-based approach described here is also the only one that allows for the purification of myogenic progenitors at different steps of their lineage progression. Taking advantage of a set of mutant mice expressing a reporter under the control of the regulatory sequences of genes explicitly expressed during specific phases of the maturation of developmental progenitors (Table 1), it is possible to purify individual myogenic progenitors that, although coexist in the same environment, present differences in terms of commitment and differentiation.

**Table 1:** Summary of the mouse lines described in this protocol.

**Genotype**	**Genetic manipulation**	**Tested**	**Comments**	**References**
*Myf5*^*GFP*−*P*/+^	Genetic knock-in: a sequence encoding eGFP replaces part of exon 1 of *Myf5*.	E/F	Myf5 is the first of the determination MRFs to be expressed in myogenic progenitors throughout development.	Kassar-Duchossoy et al., 2004
*Pax3*^*GFP*/+^	Genetic knock-in: a sequence encoding eGFP replaces exon 1 of *Pax3*.	E/F	Expressed in embryonic progenitors before myogenic determination. GFP expression persists in myogenic progenitors at fetal stages (Relaix et al., 2005).	Relaix et al., 2005
*Myf5-NN^*Cre*/*wt*^; Z/RED^*mut*/*wt*^*	Genetic mutant: a sequence encoding for Cre recombinase is inserted in the 3'UTR of *Myf5*. The *MC1-Neo* cassette was removed.	E (limbs)	Relies on a Cre recombinase-inducible reporter such as the *Z/RED* transgenic line (Vintersten et al., 2004). Possible ectopic Cre-mediated reporter expression (Figures 4A,B).	Haldar et al., 2008
*MCre^*mut*/*wt*^; Z/RED^*mut*/*wt*^*	Transgenic expression of Cre-recombinase under the control of hypaxial muscle element of *Pax3*.	E (limbs)	Muscle specific, expressed only in hypaxial embryonic precursors. Variable efficiency (Schienda et al., 2006). Relies on a Cre recombinase-inducible reporter (Vintersten et al., 2004). Potential ectopic Cre-mediated reporter expression.	Brown et al., 2005
*Pax7-ICN^*Cre*/*wt*^; Z/RED^*mut*/*wt*^*	Genetic mutant: a sequence encoding for Cre recombinase is inserted in the 3'UTR of *Pax7*.	F (limbs)	Pax7 is expressed in undifferentiated myogenic progenitors at embryonic and fetal stages in the trunk and at fetal stages in the limbs. Relies on a Cre recombinase-inducible reporter (Vintersten et al., 2004). Possible rare ectopic Cre-mediated reporter expression (Figure 4B).	Keller et al., 2004

*E, embryonic stage; F, fetal stage*.

### Experimental design

The isolation protocol consists of two main parts: (i) the isolation and identification of the fluorescent progeny (Steps 1–14) and (ii) the dissection of embryos or fetuses, followed by the enzymatic dissociation of cells and FACS isolation of progenitors (Steps 15–32). We also describe how to freeze the cells for different types of analysis and how to culture them *in vitro* (Step 33). The execution of this protocol generally requires the involvement of an animal husbandry and a cytometry facility. Operators need minimal manual skills, basic knowledge of mouse embryology and anatomy, tissue culture and cytofluorimetry.

#### Obtaining the fluorescent embryos or fetuses

The protocol we describe here is based upon mutant mice that express a reporter gene in developmental myogenic progenitors. Over the years, we gained experience with five mouse strains and tested their suitability to isolate progenitors at specific developmental stages and from specific anatomical districts (Table 1). The mice we use rely on the regulatory sequences of *Pax* specification and *Myf5* determination genes for the induction of the reporter gene (*eGFP or DsRed.T3*). These strains allow the purification of myogenic progenitors with a different stage of commitment or differentiation. Pax3 is expressed in muscle progenitor cells that subsequently become myogenic and form skeletal muscle. The commitment to the myogenic program is dependent on the expression of a determination gene belonging to the MRF family, such as *Myf5* (Figure 2A). Therefore pure population of embryonic (and fetal) committed myoblasts can be isolated from *Myf5*^*GFP*−*P*^ mice, whereas a pool of uncommitted (Myf5^−ve^) and committed progenitors can be isolated from *Pax3*^*GFP*^ mice (Figure 2B; Biressi et al., 2008). *Pax3*^*GFP*^ mice are suitable to enrich for undifferentiated myogenic progenitors also at fetal stages. Indeed due to its stability, eGFP is reportedly expressed in uncommitted fetal progenitors even though the *Pax3 locus* is downregulated (Relaix et al., 2005). We also differentially enriched for embryonic committed and uncommitted progenitors, by employing alternative mouse strains expressing Cre recombinase under the control of the regulatory regions of *Pax3* and *Myf5* (Table 1 and Supplementary Figure 1A; Boutet et al., 2010). *Pax7-ICN*^*Cre*/*wt*^ mice expressing Cre from the *Pax7 locus* can conversely be effectively used to purify myogenic progenitors from fetuses (Table 1 and Supplementary Figure 1A). These approaches depend on the conditional expression of a reporter under the control of a constitutive promoter upon the induction of Cre recombinase. We regularly used the *Z/RED* construct for expressing the reporter *DsRed.T3* from a transgene comprising a *CMV enhancer* and *chicken* β*-actin promoter* upon Cre-dependent activation (Vintersten et al., 2004), but alternative reporter lines such as those relying on the *ROSA26 locus* provide an alternative (Soriano, 1999; Abe and Fujimori, 2013). Noteworthy, the described strategies based on the Cre-LoxP system and those dependent on the direct expression of the reporters from the locus of the myogenic genes display some intrinsic differences that should be taken into account before extrapolating and comparing conclusions from data obtained with one or the other approach. Indeed, upon Cre-mediated recombination, the expression of the reporter will become independent from the regulatory sequence controlling Cre expression, and due to its dependency on a constitutive promoter will remain active also when mononucleated progenitors fuse into the fibers. Moreover, the nature of strains expressing Cre and the time required for Cre to be expressed and effectively activate the expression of the reporter gene may potentially determine a delay in the expression of the reporter compared to the expression of the endogenous marker (Supplementary Figure 1B). Beside the five mouse strains that we describe here, several other lines have been reported, which express reporter genes or Cre recombinase under the control of the regulatory sequences of muscle-characteristic genes (Tallquist et al., 2000; Chen et al., 2005; Engleka et al., 2005; Haldar et al., 2007; Bosnakovski et al., 2008; Sambasivan et al., 2009; Wood et al., 2013; Southard et al., 2014). Some of them have the advantage of being commercially available from Jackson labs. Although they require validation, they are likely to be suitable for developmental myogenic progenitor purification with this protocol.

The involvement of distinct classes of progenitors in successive phases of myogenesis, calls for accurate identification of the developmental stages. Some instructions to set up timed pregnant females are provided for the investigators that are approaching developmental studies for the first time, along with some advice on the breeding strategies we use to facilitate the successful completion of the protocol. Along the same line, a detailed description of the steps required to rapidly collect viable embryos and fetuses is also provided. Moreover, we detail in this protocol the modality of selection that is applied to immediately identify embryos and fetuses that express the reporter in the myogenic compartment. Although genotyping protocols that require a relatively short time are available (and may still be performed as a verification step), the possibility to immediately identify embryos and fetuses that are suitable for progenitor isolation is crucial to ensure the viability of the purified cells. In this protocol, we also discuss some limitations of the mouse strains we use and offer some tips to overcome them.

#### Dissociation and FACS isolation

Myogenic progenitors represent only a small fraction of the cells present in the embryonic and fetal mesoderm. To increase the yield of myogenic progenitors, and reduce the time required for their isolation, it is imperative to collect the portions of the embryos and fetuses, which are particularly enriched for myogenic progenitors, eliminating those devoid of myogenic progenitors. The body organization dramatically changes as the developing organism transits from the embryonic to the fetal stage. We, therefore, describe two alternative approaches respectively applicable to E10.5–E12.5 embryos (Step 14A) and to E14.5–E17.5 fetuses (Step 14B). This phase of dissection is crucial also to eliminate the portion of the embryos/fetuses in which the reporters are expressed outside the myogenic compartment. Indeed, the *Pax3, Pax7*, and *Myf5 locus* are reportedly active also in the developing nervous system (including neural tube and dorsal root ganglia; Tajbakhsh et al., 1994; Tajbakhsh and Buckingham, 1995; Buckingham and Relaix, 2015). Moreover, a number of observations suggest that cells with a history of Myf5 expression contribute to fetal rib chondrogenesis and brown adipose tissue formation. A contribution of cells of the Myf5-lineage to the pool of dermal precursors in the embryonic epaxial domain has also been proposed (Haldar et al., 2008). Therefore, although we have regularly used the *Myf5-NN*^*Cre*^ line to effectively purify myogenic progenitors from the limbs at embryonic stages (Boutet et al., 2010; Biressi et al., 2013), we recommend the assessment of the expression of myogenic markers before extending the protocol to different stages and portions of the embryos. Similarly, we used the *Pax7-ICN*^*Cre*^ line to successfully purify myogenic progenitors from fetal limbs (Biressi et al., 2013), but the report of an active *Pax7 locus* in progenitors contributing to dermal and brown adipose lineages at embryonic stage put in question the possibility to use the same approach in different conditions (Lepper and Fan, 2010). For each genotype described in this protocol, the validated developmental time and body domains are indicated in Table 1.

Embryonic and fetal tissues are composed of multiple types of cells that are continuously interacting with each other and with the extracellular matrix. As development proceeds, multinucleated fibers appear in the muscle anlagen. An efficient release of mononucleated cells is crucial to maximize the yield of myogenic progenitors. In this protocol, dissected embryos and fetuses are subjected to cycles of physical and enzymatic dissociation, under buffered conditions. A filtration step will ensure the removal of fiber debris.

The cellular suspension is immediately processed by FACS. When the dissection and isolation phases have been optimized, and the appropriate controls used, populations of cells expressing or not expressing the reporter can be readily identified (**Figure 7** and Supplementary Figure 4). Defining appropriate SSC and FSC gates is crucial to eliminate dead cells and debris, in particular at the fetal stage when more mature muscle fibers are present. With experience, the great majority of isolated cells are viable, but the addition of live-dead dyes, such as 7-AAD (7-amino-actinomycin D) or propidium iodide may help in identifying them and verify that the procedure has been performed correctly (Biressi et al., 2007b).

#### Characterization of purified myogenic progenitors

Myogenic progenitors isolated with this protocol can be processed for molecular investigations, such as the quantification of transcript or protein levels, which require RNA and protein extraction, respectively. Furthermore, cells can be cultured *in vitro* to evaluate intrinsic parameters such as proliferation, motility, differentiation, and fusion into multinucleated syncytia with different morphological, biochemical and functional properties (Biressi et al., 2007b; Boutet et al., 2010). For investigators learning this protocol or adapting it to new conditions and developmental stages, we recommend to verify the purity of the sorted populations by reanalyzing a small number (>1,000) of cells at the cytofluorimeter (Supplementary Figure 5) or by plating them and stain them with antibodies recognizing the reporter used for isolation or myogenic markers (**Figure 8**).

## Materials and equipment

### Reagents

- *WT* and mutant mice expressing a reporter in myogenic progenitors (Table 1). We have used *WT* CD1 or C57BL/6 mice from the Jackson Laboratory or Charles River. **CAUTION** Experiments using murine strains must be approved by the Institute Animal Welfare Body and by the local authorities, and must conform to EU law of the Member countries (2010/63/UE) or relevant national and institutional regulations and laws.
- 1x phosphate buffer saline (PBS) pH 7.4 (ThermoFisher Scientific 10010031)
- Ethanol 70% (vol/vol)
- DMEM (High Glucose) (ThermoFisher Scientific 10938025)
- Penicillin/streptomycin mixtures (P/S) (100X, Omega Scientific PS-20)
- HEPES 1M (ThermoFisher Scientific 15630080)
- L-Glutamine 200 mM (ThermoFisher Scientific 25030081)
- 1x Ca^2+^-free Hanks' balanced salt solution (HBSS) (ThermoFisher Scientific 14170070)
- Bovine serum albumin (BSA) (Sigma A1933)
- Glucose (Sigma G7021)
- Magnesium sulfate (MgSO_4_) (Sigma M2643)
- Sodium hydroxide (NaOH) 1N (Sigma S2770)
- EDTA disodium salt (Sigma E6635)
- Ammonium chloride (NH_4_Cl) (Sigma A9434)
- Potassium bicarbonate (KHCO_3)_ (Sigma 60339)
- EDTA tetrasodium salt hydrate (Sigma E5391)
- Fetal bovine serum (FBS) (ThermoFisher Scientific 10270106)
- Collagenase Type V (Sigma C9263)
- Dispase (ThermoFisher Scientific 17105041)
- DNAse I (Worthington LS002139)
- Parafilm
- Horse serum (ThermoFisher Scientific 16050122)
- Calf skin collagen (Sigma C8919)
- ECM gel from Engelbreth-Holm-Swarm murine sarcoma (Sigma E1270)
- Ultrapure distilled water (ThermoFisher Scientific 10977023)
- 16% Formaldehyde (w/v), Methanol-free (ThermoFisher Scientific 28906)
- CAUTION Hazardous in case of eye contact and on ingestion
- Anti-Pax7 (Developmental Studies Hybridoma Bank AB_528428)
- Anti-MyoD (Dako M3512)
- Anti-Myogenin (BD Pharmingen 556358)
- Anti-Desmin (Sigma SAB5600054)
- DAPI (diamidino-2-phenylindole) (Sigma 32670)
- Ice
- Liquid nitrogen.

### Equipment

- 10-cm Petri dishes
- 37°C shaking water bath (Grant LSB18 or equivalent)
- CO_2_ chamber
- Dissection scissors
- Dumont forceps with curved tips
- Dumont forceps with straight tips
- Inverted fluorescent microscope
- Sterile hood for cell culture
- Stereomicroscope
- 6-well culture dish
- Microscope glass slides (ThermoScientific J1800AMNZ or equivalent)
- Permanent Marker
- Insulin syringes with G27 needles
- Sterile 500-ml beaker
- Magnetic stirrer
- PH-meter
- Stericup Filter Units with 0.22-μm filters (Millipore SCGVU02RE)
- Steriflip Vacuum Driven Sterile Filters with 0.22-μm filters (Millipore SCGP00525)
- 15-ml conical tube (Falcon 352096 or equivalent)
- 50-ml conical tubes (Falcon 352070 or equivalent)
- Weights for water bath
- Racks for 15-ml and 50-ml conical tubes
- Sterile 5-ml serological pipets and a pipet controller
- Refrigerated microcentrifuge (Sorvall Legend Micro 21R or equivalent)
- Refrigerated centrifuge with swing rotor (Eppendorf 5810R or equivalent)
- Falcon 5-ml round bottom tubes with strainer cap (Fisher Scientific 08-771-23 or equivalent)
- Falcon 5-ml round bottom tubes (Fisher Scientific 14-959-2A or equivalent)
- Cell counting chamber (Hemocytometer)
- BD FACSAria II or Vantage SE cell sorter (BD Biosciences)
- 1,5-ml conical bottom microcentrifuge tube
- BioCoat™ Poly-D-Lysine 8 Well Culture Slides (Corning 354632)
- 96-well culture dish
- Standard CO_2_ cell culture incubator
- 2–20, 20–200, 100–1,000 μl Gilson pipets and matching filtered sterile tips
- Examination gloves
- Ice bucket.

### Reagents setup

**Supplemented DMEM medium** DMEM (High Glucose) is supplemented with sterile P/S (1x), glutamine (2 mM), HEPES (10 mM). It is recommended that renew glutamine every couple of weeks. Store the medium at 4°C until ready to use.

**Dissociation Buffer** This solution is consisting of HBSS containing 0.3% (weight/vol) BSA, 15 mM HEPES, 1.5 mM MgSO_4_ and 15 mM glucose. The pH of the solution is set to 7.4 with NaOH by using a pH-meter. The sterility of the solution is obtained by filtering through 0.22-μm Stericup filters. This solution may be stored at 4°C for a month.

**Stock Collagenase/Dispase Solution** Dissolve Collagenase V and Dispase powder in 1X HBSS so that the final concentrations are 3.75 mg/ml and 12 U/ml, respectively. Sterilize with 0.22-μm Steriflip filters and store as 1-ml aliquots at −20°C. Thaw before use.

**Stock DNAse I Solution** Reconstitute the lyophilized powder in sterile water at the final concentration of 10 mg/ml. Store as 500-μl aliquots at −20°C. Thaw before use.

**Digestion Solution** This reagent should be prepared freshly by dissolving Stock Collagenase/Dispase Solution (3%, vol/vol) and Stock DNAse I Solution (0.1% vol/vol) in Dissociation Buffer, and kept on ice until needed. Prepare 8 or 16 ml every 6 embryos or fetuses, respectively.

**Erythrocyte Lysis Solution** Dissolve in 1x PBS NH_4_Cl (0.15 M), KHCO_3_ (10 mM), EDTA tetrasodium salt hydrate (0.1 mM) and sterilize with 0.22-μm Steriflip filters. This solution may be stored at room temperature for several weeks.

**Sorting Solution** Dilute supplemented DMEM medium with 20% (vol/vol) FBS in a beaker. On a magnetic stirrer dissolve EDTA disodium salt (2 mM). Sterilize the resulting solution with 0.22-μm Stericup filters. Store the medium at 4°C until ready to use.

**Collecting Medium** Dilute supplemented DMEM medium with 50% (vol/vol) FBS. Store the medium at 4°C until ready to use.

**Plating Medium** 20% (vol/vol) horse serum is added to supplemented DMEM medium. Store the medium at 4°C until ready to use.

**Collagen coating Solution** This solution is prepared by diluting the commercial solution (1 mg/ml) 10-fold with sterile water.

**ECM coating Solution** This solution is prepared by diluting ECM gel 1:100 in ice-cold non-supplemented DMEM. Prepare freshly before use. It is important to keep the reagents constantly at 4°C as ECM gel solidifies at a higher temperature.

### Equipment setup

**BD FACSAria cell sorter** The 488 nm laser is required for the detection of the fluorochromes recommended in this protocol. We use a BD FACSAria II sorter, but the scheme we describe here should be applicable also to other models. Calibrations are verified with tracking beads, and the presence of a stable breakoff is ensured according to the manufacturer's guidelines.

**Collagen-coated plastic cell culture dishes** Cover cell culture plastic wells with collagen coating solution. Allow the collagen to bind for several hours at room temperature or overnight at 4°C. Carefully aspirate excess fluid and allow it to dry completely in a cell culture hood. Overnight exposure to UV light in a cell culture hood may ensure sterility. Collagen-coated wells may be sealed with Parafilm and stored for a few weeks at room temperature.

**ECM-coated glass chamber slides** BioCoat™ Poly-D-Lysine 8 Well Culture Slides should be coated with freshly prepared ECM coating solution. Add a sufficient volume of freshly prepared ECM coating solution to cover the bottom of the vessels. Incubate for a minimum of 6 h at 4°C. The culture slides can then be stored at 4°C for up to a week with the coating solution on them. Aspirate the coating solution and seed cells immediately.

## Procedure

### Setting up timed pregnant mice-TIMING 5 min

1. Add 1-2 females to each stud male cage. As females will be sacrificed during the following steps of the procedure, we usually cross mutant males with commercially available *WT* females. This strategy has some limitations that are discussed in Step 14 of this protocol.
2. Check the presence of vaginal plugs the subsequent morning. We consider the morning a plug is observed as gestational day E0.5.
  **CRITICAL STEP** The presence of a vaginal plug does not necessarily guarantee pregnancy, but only indicate that mating occurred. In our experience, the likelihood of pregnancy after mating depends on the mouse strain. When the experimental design does not require the use of mice of a specific background, we use the outbred CD1 strain that has higher fertility, compared to the classically used C57BL/6 strain.

### Collection of embryos and fetuses-TIMING 20 min per pregnant female

3. Prepare 10-cm Petri dishes with 10 ml of sterile PBS (four dishes per pregnant female). PBS should be pre-warmed at 37°C and dishes containing PBS should maintained at 37°C until their use.
4. Euthanize the pregnant female in a CO_2_ chamber and subsequently perform cervical dislocation to ensure death.
  **CAUTION** The method used to kill mice must be approved by your institutional guidelines and national laws.
5. Spray the skin of the mouse with 70% (vol/vol) ethanol, and place it in the dissection area.
6. Make a U-shaped incision of the abdomen (skin and muscle layers) with scissors (Supplementary Figure 2A).
7. Lift the abdominal wall up with forceps to expose the uterus (Supplementary Figure 2B).
8. Grasp the uterine horns close to their convergence at the cervix with forceps, and cut them free with fine scissors (Supplementary Figure 2C).
9. Carefully pull the complete uterus out of the abdominal cavity with fine forceps and place it in a dish containing 10 ml of PBS (Supplementary Figure 2D).
10. After a couple of minutes, gently transfer the uterus in a clean dish containing PBS to wash the surrounding blood away.
11. With fine scissors and forceps open the uterus to expose the embryos/fetuses enclosed in their yolk sac (Supplementary Figures 2E,F).
  **CRITICAL STEP** Be careful not to squeeze or cut the embryos.
12. With fine sterile forceps remove each embryo/fetus with yolk sac from the placenta and gently transfer it to a clean dish containing PBS to eliminate the surrounding blood (Supplementary Figures 2G,H).
13. Gently remove the yolk sac (Supplementary Figure 2I**)** and the amniotic sac (Supplementary Figure 2J), and transfer the embryos/fetuses in a dish containing PBS.

### Identification of fluorescent progeny -TIMING 20 min per pregnant female

14. When we are setting up the breeding (Step1), we usually use heterozygous mutant stud males. This is an obvious requirement for strains that may be lethal in homozygousity, such as the *Pax3*^*GFP*^ strain, which is devoid of Pax3 expression. The implication of this approach is the presence of both “fluorescence positive”(mutants expressing the fluorescent reporter required for the identification of the myogenic progenitors) and “fluorescence negative” (used as negative controls in Step 28) progeny. To finalize the protocol in a timely manner and to optimize the yield of myogenic progenitors, a key point is to identify the fluorescent embryos before the dissection and the enzymatic digestion (Steps 15-22). The modality of selection of the fluorescent progeny is different according to the developmental stage: embryonic stage (from E10.5 to E12.5) (option A) or fetal stage (from E14.5 to E17.5) (option B). Both options imply the identification of the progeny expressing the fluorescent reporter in the myogenic compartment by microscopic investigation. These approaches are:

### (A) Selection of fluorescent embryos

**Figure 3:**
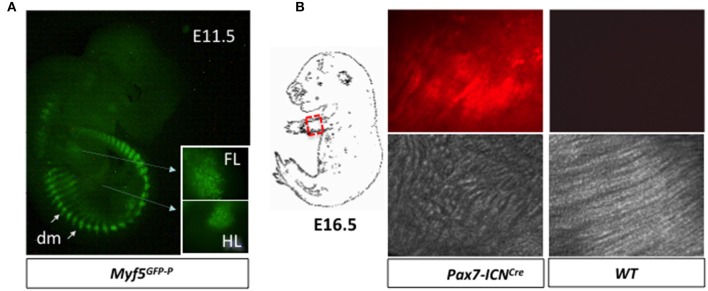
Identification of embryos and fetuses suitable for progenitor purification. **(A)** Green fluorescence in the myotome/dermomyotome (dm), fore (FL), and hind (HL) limbs of an E11.5 *Myf5*^*GFP*−*P*/+^ embryo. **(B)** Limbs of E16.*5 Pax7-ICN*^*Cre*/*wt*^*; Z/RED*^*mut*/*wt*^ and *WT* fetuses (dashed line) were squeezed between two microscope slides, and red fluorescence was evaluated. Note that muscle fibers, which are clearly apparent in the bright field (lower), are fluorescent only in *Pax7-ICN*^*Cre*/*wt*^*; Z/RED*^*mut*/*wt*^ fetuses (upper).

- Prepare a 10-cm Petri dish with 10 ml of sterile PBS. PBS should be pre-warmed at 37°C.
- Place the dish containing embryos under an inverted fluorescence microscope and observe with the filter appropriate for the employed reporter (Figure 3A).
  **CRITICAL STEP** The selection of the fluorescent embryos must be performed as fast as possible to improve the viability of their cells. Working under sterile conditions and wearing examination gloves will decrease the chances of contamination.
  **CRITICAL STEP** In our experience, the skin is sufficiently transparent before E12.5 to readily allow the identification of the fluorescent progeny in most of the fluorescent microscopes. Ultrafast genotyping protocols on the yolk sacs of the individual embryos may be used as an alternative (Lopez, 2012). Moreover, genotyping may be employed to confirm the microscopic evaluation when the experiment is initially attempted or if it is adapted to murine lines different from those detailed in this protocol.
- With sterile forceps transfer the embryos presenting the correct pattern of reporter gene expression in the myogenic compartment in a clean dish containing supplemented DMEM medium. Set also some non-fluorescent embryos apart to use them as negative controls during the cytometer isolation (Step 28).
  **CRITICAL STEP** Be careful not to damage the body of the embryo. The use of curved forceps should allow to gently grabbing the embryos without piercing them. Alternatively pinch portions of the embryo that will be subsequently dissected away, such as the head (Step 15).

### (B) Selection of fluorescent fetuses

**Figure 4:**
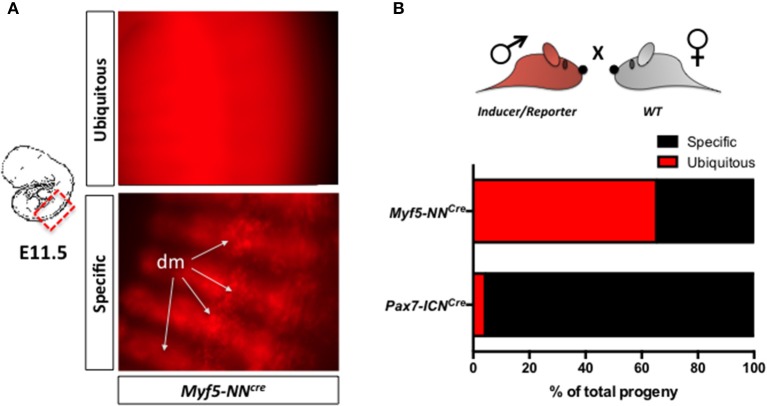
Occasional ectopic Cre-mediated reporter expression in compound mutant. **(A)** Representative pictures of the trunk of *Myf5-NN*^*Cre*^*; Z/RED*^*mut*/*wt*^ E11.5 embryos presenting ubiquitous (upper) or muscle-specific (lower) fluorescence. In the latter case, DsRed.T3^+ve^ myotomes/dermomyotomes are easily identified at the microscope. **(B)** Quantification of the progeny characterized by muscle-specific or ubiquitous reporter gene expression after crossing double mutant males presenting both “inducer” (expression of Cre recombinase) and “reporter” (Cre-mediated permanent expression a reporter gene) features with *WT* females. *Myf5-NN*^*Cre*^ and *Pax7-ICN*^*Cre*^ inducer lines are compared to each other.

- Prepare 6-well dishes and fill each well with 3 ml of supplemented DMEM medium pre-warmed at 37°C. With a permanent marker label each well with a number. The total number of wells should match the number of fetuses that have been collected.
- With forceps move each fetus in an individual well.
- With fine forceps remove one of the forearms from each fetus, dissect away the hand, remove the skin, and squeeze it between two glass slides. Mark the slides with the number of the corresponding fetuses.
  **CRITICAL STEP** Be careful that the tissue between the glass slides is not drying out, as it will increase auto-fluorescence. We usually do not process more than 1-2 fetuses at the same time.
- Place the glass slides under a fluorescence microscope and observe with the appropriate filter to identify fluorescent and non-fluorescent fetuses (Figure 3B).
  **CRITICAL STEP** It is important to vigorously squeeze the tissue between the two slides until it forms a thin layer. If this step is not performed properly, the auto-fluorescence of the tissue may become confounding. Auto-fluorescence is particularly high in bones and in possibly remaining parts of the skin. This could be misleading, in particular when the protocol is attempted the first time. Before analyzing the pattern of reporter gene expression, we suggest to focus in the bright filed on the muscle mass, which is easily identifiable by the presence of fibers at fetal stage (Figure 3B). The direct comparison with non-fluorescent fetuses may also be helpful (Figure 3B).
  **CRITICAL STEP** *Cre* transgenes hold the potential to induce efficient recombination of *loxP* sequences in the germline (Schmidt-Supprian and Rajewsky, 2007). This is particularly frequent in the female germline but may also occur in the male (Rempe et al., 2006; Shi et al., 2016). This is particularly relevant when mutant mice that are used to mark myogenic progenitors, are double compound mice in which an allele is driving the expression of Cre-recombinase in the myogenic compartment (inducer allele), and another is permanently expressing a reporter gene upon Cre-dependent induction (reporter allele) (Abe and Fujimori, 2013). We regularly employ three distinct inducer lines to isolate myogenic progenitors (Table 1, Supplementary Figure 1A). We usually cross males carrying both the inducer and the reporter allele with *WT* females (Step 1), and we regularly observe that, although a large fraction of the progeny is correctly expressing the reporter in the myogenic lineage, in a variable fraction of the progeny the reporter is diffusely expressed outside the myogenic compartment (Figure 4A, Supplementary Figure 3). Importantly, this phenomenon occurs at different rates in distinct inducer lines, being the *Myf5-NN*^*Cre*^ line particularly affected (Figure 4B). This problem requires a careful microscopic evaluation not only of the presence of the fluorescent reporter but also of its pattern of expression. Only embryos or fetuses specifically expressing the reporter in the muscle lineage should be further processed to obtain pure populations of myogenic progenitors.

### Dissection of embryos and fetuses -TIMING 5-10 min per embryo/fetus

15. To optimize the purity and yield of myogenic progenitors, and reduce the time of the procedure, it is important to eliminate portions of embryos and fetuses in which myogenic progenitors are scarce or absent. The modality of dissection is different according to the developmental stage: embryonic stage (from E10.5 to E12.5) (option A) or fetal stage (from E13.5 to E17.5) (option B).

### (A) Dissection of embryos

**Figure 5:**
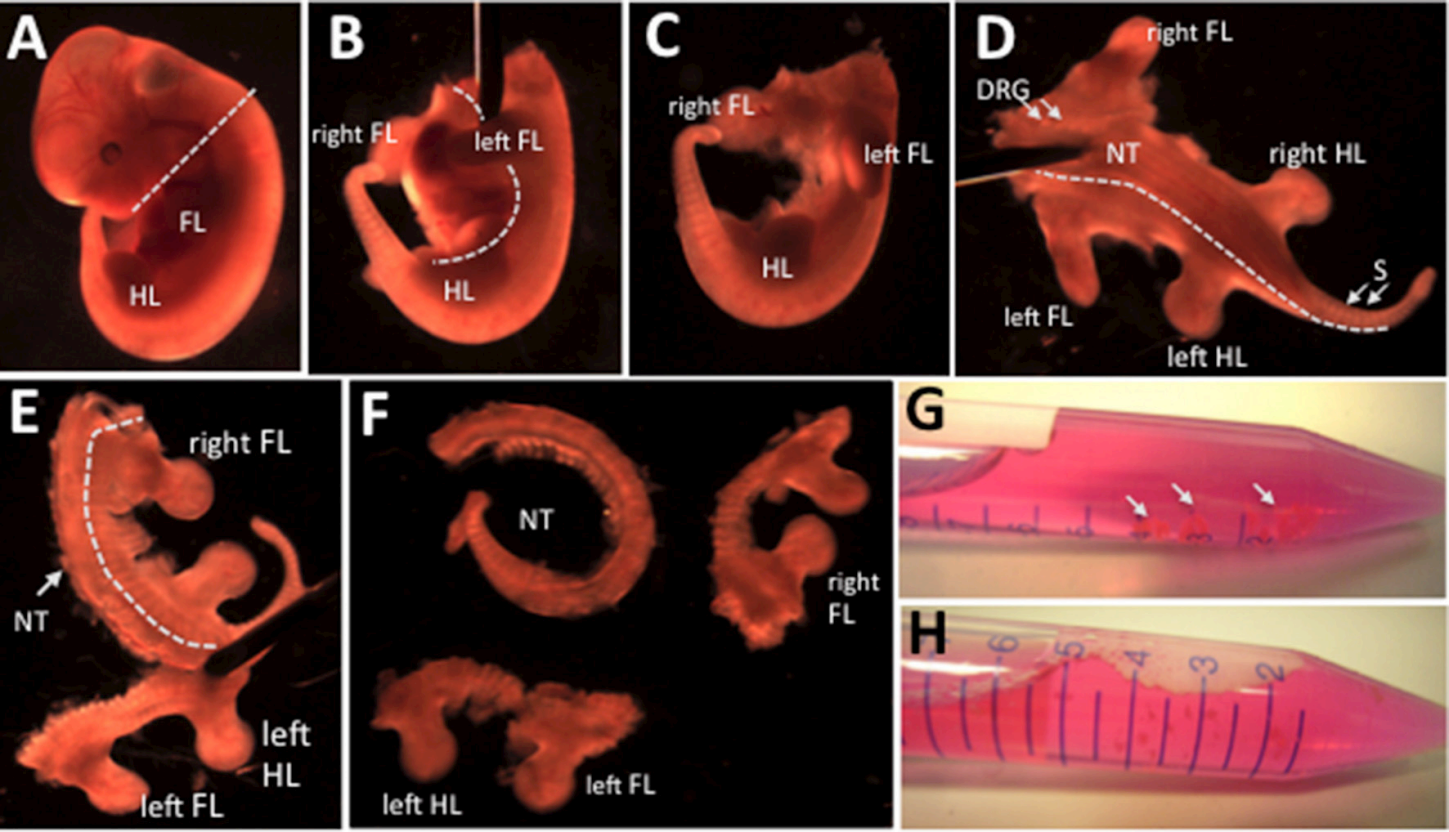
Progressive dissection of an E11.5 embryo. **(A)** The head of the embryo is removed. **(B)** After placing the embryo on one side, the internal organs laying between the upper (FL) and lower (HL) limbs are removed by using needles. **(C)** Photo of an E11.5 embryo after removal of the internal organs presenting intact FL, HL, and lateral body wall. **(D)** After placing the embryo with the ventral portion facing down, needles are used to cut the embryo cranio-caudally (dashed line) lateral to the dorsal root ganglia (DRG) and neural tube (NT) and medial to the somites (S). **(E)** The operation described in **(D)** is repeated on both sides of the NT. **(F)** The embryo (divided into a right and left portion) is dissected free from the NT. **(G)** The isolated portions the embryos are placed in a 15 ml tube containing digestion solution. **(H)** Through vigorous shacking embryonic tissues are disaggregated in small (<1 mm^3^) pieces.

- Prepare two 10-cm Petri dish with 10 ml of sterile supplemented DMEM medium. DMEM should be pre-warmed at 37 °C.
- Dissect away the head (Figure 5A) and the internal organs (Figure 5B) of each embryo. We use syringes with needles to both hold and cut the embryos. To dissect we use the oblique part of the needle as a blade.
  **CRITICAL STEP** We suggest performing this step under a stereomicroscope to guarantee a clean removal of the internal organs without damaging the rest of the embryo. This will facilitate the subsequent steps of the dissection.
- With forceps transfer the embryos free from the internal organs (Figure 5C) in a clean dish containing supplemented DMEM medium, and position them with the ventral portion facing down, in a “lion's skin” position (Figure 5D). This operation should be separately done both for fluorescent and non-fluorescent (control) embryos.
  **CRITICAL STEP** Be careful not to damage the body of the embryo. The use of curved forceps should allow to gently grabbing the embryos without piercing them.
- Under a stereomicroscope cut the embryos lateral to the neural tube and the chain of dorsal root ganglia. Use a syringe needle to hold the embryo in position and with the other cut on both side of the neural tube (Figures 5D,E) in order to completely separate the neural tube with the associated dorsal root ganglia from the rest of the body (Figure 5F).
- Transfer the embryos free from the neural tube in a 15-ml tube, containing the Digestion Solution (Figure 5G) and shake vigorously by hand 2-3 times to disaggregate the embryos in small pieces (Figure 5H).
  **CRITICAL STEP** If the experimental design is requiring that myogenic progenitors should be isolated from specific portions of the embryos, such as the limb buds, those portions should be further dissected at the stereomicroscope, and only those should be transferred in the Digestion Solution.
  **CRITICAL STEP** The volume of Digestion Solution should be sufficient to allow for effective physical dissociation of the embryos during the enzymatic digestion. We usually fill each15-ml tubes with ~8 ml of Digestion Solution with not more than 6 embryos. Multiple tubes may be required if multiple litters are processed at the same time. In general, we do not suggest to process more than a dozen embryos, as it will slow the isolation procedure affecting cell viability.

### (B) Dissection of fetuses

**Figure 6:**
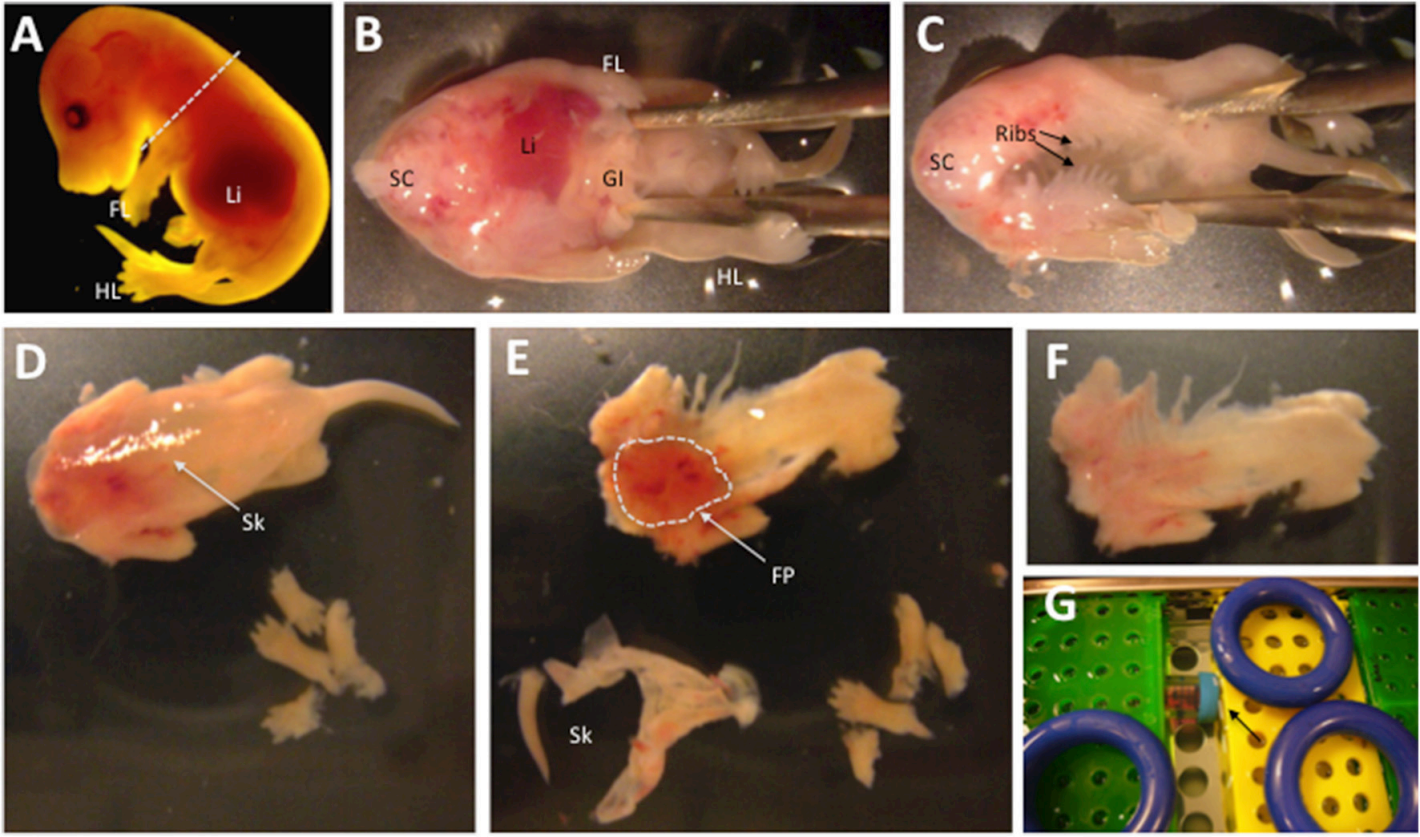
Progressive dissection of an E15.5 fetus. **(A)** The head of the fetus is removed. **(B)** After placing the fetus with the ventral portion facing up, forceps are used to cut the thorax and abdomen and expose the internal organs. **(C)** The fetus shows an empty body cavity after removal of the heart, lungs, liver, gastrointestinal tract and other internal organs. **(D)** E15.5 fetus with the dorsal portion facing up, from which limb extremities have been dissected out. The skin (Sk) is translucent and clearly visible. **(E)** A skinned fetus presenting large inter-scapular fat pads (FP, dashed line). **(F)** A skinned fetus from which inter-scapular fat pads have been removed. **(G)** Position of the tubes (arrow) in the water bath during enzymatic digestion. Additional abbreviations in the scheme: Li, Liver; FL, fore limes; HL, hind limbs; SC, spinal cord; GI, gastrointestinal tract.

- Prepare two 10-cm Petri dish with 10 ml of supplemented DMEM medium. DMEM should be pre-warmed at 37°C.
- Dissect away with forceps the head of the fetuses and transfer the fetuses devoid of the head in a clean dish containing supplemented DMEM medium (Figure 6A). Fluorescent and non-fluorescent (control, Step 28) fetuses should be pooled in different dishes.
- Place each fetus with the dorsal portion facing down and with forceps cut longitudinally the rib cage and the abdomen to expose the internal organs (Figure 6B).
- Eviscerate each fetus with curved forceps and transfer it in a clean dish containing supplemented DMEM medium (Figure 6C). The separation between fluorescent and non-fluorescent (control) fetuses should be maintained.
- Cut the extremities of the fore and hind limbs, leaving the forearm and crus muscle intact. Position the fetus with the ventral portion facing down, and with forceps remove the skin at the stereomicroscope (Figure 6D). With curved forceps, we grab the skin anteriorly, and by holding the underneath tissues with other forceps, we pull it up caudally until reaching the tail (Figure 6E).
  **CRITICAL STEP** It is crucial to carefully remove the extremities to obtain complete removal of the skin. Whereas at E15.5 or later stages the skin can generally be removed in a single piece, before E15.5 it consists in a thin layer that may require a careful elimination of remaining pieces at the stereomicroscope.
- With curved forceps carefully eliminate the interscapular fat pads from the skinned fetuses (Figures 6E,F).
  **CRITICAL STEP** If the experimental design is requiring that myogenic progenitors should be isolated from specific portions of the fetuses, such as the limbs, only those portions should be isolated from the rest of the body and processed further.
- Transfer the dissected fetal tissues in a 50-ml tube, containing the Digestion Solution. Fluorescent and non-fluorescent fetuses should be transferred in separate tubes.
  **CRITICAL STEP** The volume of Digestion Solution should be sufficient to allow for effective physical dissociation of the fetuses during the enzymatic digestion. We usually fill each 50-ml tubes with ~16 ml of Digestion Solution with not more than 6 fetuses. Multiple tubes may be required if multiple litters are processed.
  **CRITICAL STEP** The dissection must be performed as fast as possible to improve the viability of the cells. Processing more than six fetuses at the same time may slow the isolation procedure affecting cell viability.

### Isolation of mononucleated cells -TIMING 70–90 min

16. Seal the tubes well with Parafilm and position them in a shaking water bath horizontally along the shaking path. Use weights to keep the tubes completely submerged in water (Figure 6G).
17. Incubate at 37°C with agitation (~70 r.p.m. on the recommended model of shaking water bath) for 15 min.
18. Remove the Parafilm, place the tubes on a rack and let the digesting embryos or fetuses accumulate on the bottom of the tube for ~1 min.
  **CRITICAL STEP** This and the fallowing steps should be performed in a sterile hood if cells will be cultured.
19. With a 5-ml pipette gently transfer the supernatant containing the disaggregated cells in a clean labeled 15-ml tube and place it in ice.
  **CRITICAL STEP** Be careful to not aspirate the digesting embryos or fetuses. In particular, embryos form an aggregate that can easily be disturbed. To avoid it, control the aspiration strength, aspirate from the surface of the supernatant, and leave ~1 ml of supernatant in the tube.
20. Add new digestion solution to the tube containing the embryos (8 ml) or fetuses (16 ml), seal the tubes with Parafilm, shake vigorously 2–3 times by hand, and incubate as previously described for additional 15 min.
21. In the meanwhile, centrifuge the aspirated supernatant in a swinging-bucket rotor at 500 g for 5 min at 4°C. Carefully discard the solution without disturbing the cellular pellet. Resuspend the cells in ~1 ml of ice-cold Sorting Solution and place in ice.
22. Repeat Steps 18–21 until digesting pieces of tissue are not visible anymore in the case of the embryonic preparations, or until cartilage and bones are completely devoid of muscle in the case of fetal preparations. Whereas the cell pellet is initially resuspended in fresh Sorting Solution (Step 21), at the end of the subsequent cycles of digestion resuspend the cell pellet with the cell suspension obtained during the previous cycles. In this way, at the end of the digestion, the cell fractions derived from the digestion of a single pool of embryos/fetuses will end up in a single tube.
  **CRITICAL STEP** In our conditions, 3 and 4 cycles are usually sufficient to digest respectively E11.5 embryos and E15.5 fetuses. Be careful to not terminate the digestion when small undigested pieces of the embryo are still present, and to not over-digest the fetal samples, as in both cases the fraction of myogenic progenitors will be reduced.
  **CRITICAL STEP** At the fetal stage, the presence of erythrocytes may render the quantification of the cells (Step 26) and the FACS-isolation (Steps 27–32) problematic. To remove the majority of the erythrocytes we suggest to centrifuge fetal samples as described in Step 21 and resuspend the cellular pellet in Erythrocyte Lysis Buffer. After 2 min of incubation at room temperature, centrifuge the samples and resuspend with ~1 ml of Sorting Solution.
23. Transfer the cellular suspension to the strain cap attached to a 5-ml FACS tube. Pipette up and down gently to facilitate flow-through.
24. Rinse the tube that was containing the resuspended cells with another 1 ml of Sorting Solution, and transfer it to the same strainer cap.
  **CRITICAL STEP** If multiple embryos/fetuses are processed at the same time, the strainer can be clogged. Carefully recover all traces of liquid from the clogged strainer (both outside and underside) before changing to a new one.
25. Remove the strainer cap and collect with a 20–200-μl pipette any remaining liquid on the underside of the strainer. Close the tube with a plastic cap and maintain in ice.

### Cell sorting -TIMING ~20 min per embryo/fetus

**Figure 7:**
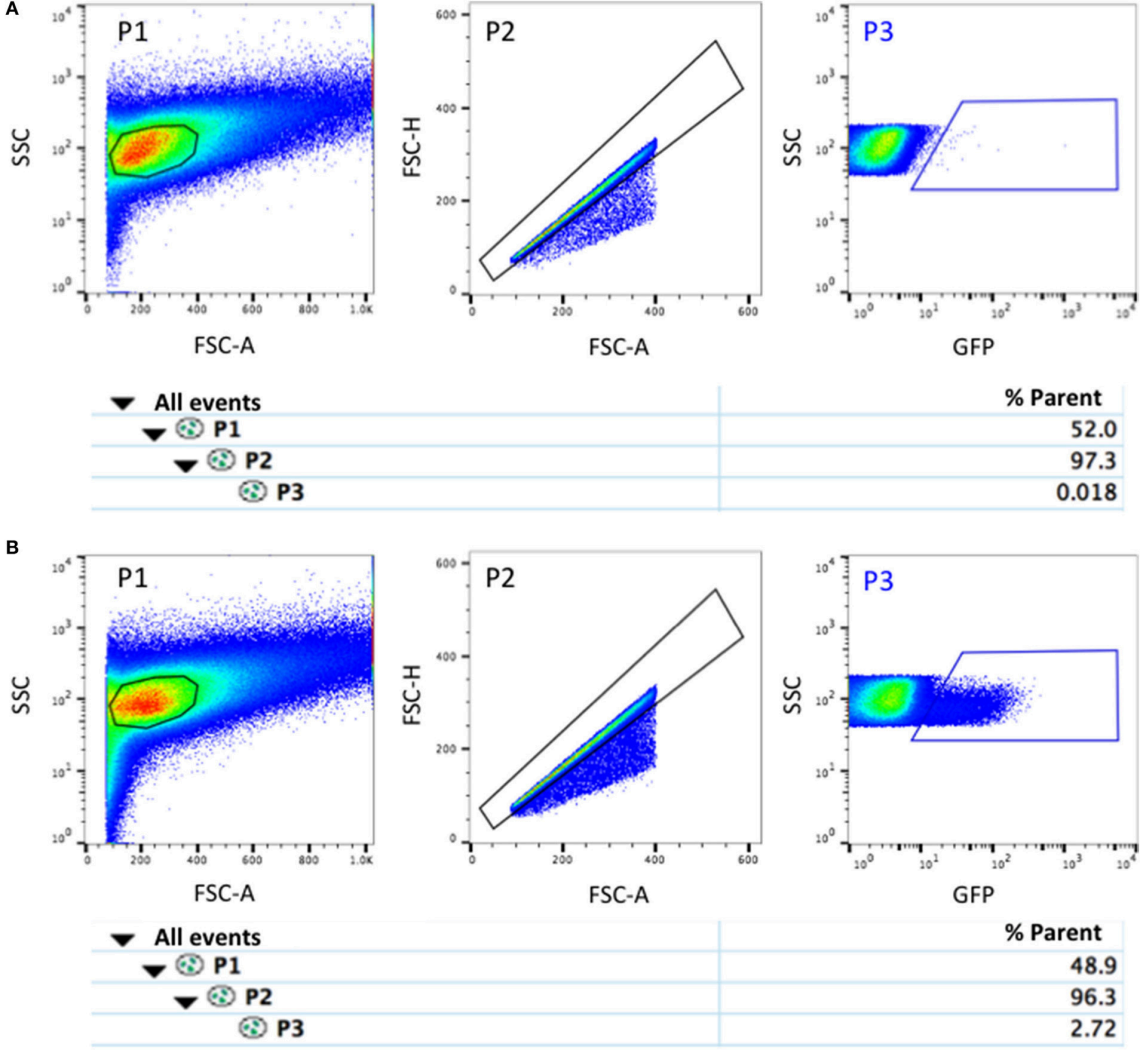
Representative FACS profiles of fetal myogenic progenitors. **(A)** Profile of cells obtained after digestion of E15.5 *WT* fetuses (GFP^−*ve*^ controls). **(B)** Profile of myogenic progenitors obtained after digestion of E15.5 *Myf5*^*GFP*−*P*^ fetuses, as described in this protocol. Cells in P3 gate are myogenic progenitors. The population hierarchy is shown under the plots. Abbreviations: SSC, Side scatter; FSC-A, Forward scatter-Area; FSC-H, Forward scatter-Height.

26. Mix the samples by gently pipetting or vortexing, estimate the concentration of the filtered cell suspension, and adjust the concentration to ~2 × 10^6^ cells per ml with ice-cold Sorting Solution. Split into multiple FACS tubes if the final volume is more than 4 ml.
  **CRITICAL STEP** It is best to move immediately to cell sorting for the highest yield and viability.
27. Set up the cell sorter according to the manufacturer's guidelines with a 100-μm nozzle.
28. Run the non-fluorescent cellular suspension to properly set the voltages. Ensure that the cell population is presenting low background fluorescence and is appropriately positioned in the SSC and FSC-A plot (Figure 7A).
29. Create a gate on the bidimensional SSC and FSC-A plot to exclude debris (P1, Figure 7).
30. Create a gate on the FSC-H and FSC-A plot to select for intact single cells (P2, Figure 7).
  **CRITICAL STEP** In our hands, this gating strategy is efficiently excluding doublets of cells, dead cells, and fragmented fibers (particularly present at fetal stage) that present an irregular shape.
31. Generate the gate to identify the fluorescently labeled cells (P3, Figure 7, Supplementary Figure 4).
32. Sort the fluorescent progenitors in a 5-ml round bottom collection tube containing 2 ml of ice-cold Collecting Media at a flow rate between 1,000 and 3,000 events per second.
  **CRITICAL STEP** We suggest to rinse the walls of the collection tube with Collecting Media to facilitate the flow to the bottom of the tube of droplets containing the sorted cells.
  **CRITICAL STEP** To reduce the risk of bacterial contamination we suggest to run 70% (vol/vol) ethanol through the fluidic system before sorting.

### Processing of sorted cells -TIMING variable

33. Sorted cells can be immediately frozen for RNA isolation (option A), for protein purification (option B), or can be plated on culture dishes or slides to follow their spontaneous differentiation (option C).

### (A) Freezing myogenic progenitors for RNA isolation -TIMING 20 min

- Transfer the content of the collection tube in a 15-ml conical tube and centrifuge in a swinging-bucket rotor at 500 g for 10 min at 4°C. With a 100–1,000-μl pipette gently remove the media down to ~1 ml, resuspend the cellular pellet and transfer it to a 1.5-ml microcentrifuge tube.
  **CRITICAL STEP** To avoid RNA degradation we suggest to use RNAse-free tubes and tips with filters.
- Centrifuge the tubes in a fixed angle rotor at 2,500 g for 5 min at 4°C and aspirate the supernatant completely with a 20–200-μl pipette.
  **CRITICAL STEP** To avoid the loss of sorted cells control the aspiration strength and aspirate from the surface of the supernatant.
- Snap-freeze the cell pellet in liquid nitrogen or by placing the tube at −80°C. Samples can be stored for several months at −80°C.
  **CRITICAL STEP** It is crucial to immediately snap-freeze the pellet to avoid RNA degradation. Dissociation in dedicated commercial buffers containing β-mercaptoethanol (i.e. RLT Buffer, Qiagen) is an alternative we successfully used.

### (B) Freezing myogenic progenitors for protein purification -TIMING 25 min

- Process cells as described in Steps 33A (i) and (ii).
- Wash the pellet by filling the tube with 1 ml of PBS, Centrifuge the tubes in a fixed angle rotor at 2,500 g for 5 min at 4°C and aspirate the supernatant completely with a 20–200-μl pipette.
- Snap-freeze the cell pellet in liquid nitrogen. Samples can be stored for several months in liquid nitrogen or at −80°C.

**CRITICAL STEP** It is crucial to proceed immediately with the freezing of the samples to avoid degradation.

### (C) *In vitro* culturing -TIMING 30–35 min to set up

- Prepare coated plastic wells or glass chambers for cell seeding.
  **CRITICAL STEP** Collagen-coated plastic wells and ECM-coated glass chambers slides can be used for culturing. Nevertheless, we suggest to use ECM-coated glass chambers for microscopic analysis after immunofluorescence.
- Plate cells in Plating Medium and place them in an incubator at 37°C with 5% CO_2_. We usually plate ~15,000 cells in 300 μl of Plating Medium per well in 8-well chamber slides or ~10,000 cells in 200 μl of Plating Medium per well in 96-well plates. After plating, cells adhere to the well surface within hours.
  **CRITICAL STEP** We usually plate cells in Plating Medium, but we successfully used also Opti-MEM (Thermofisher Scientific) supplemented with 20% (vol/vol) FBS, 20 mM HEPES, and 5 ng/ml bFGF (Peprotech) to delay terminal differentiation (Boutet et al., 2010).
- Replace half of the medium every day with fresh Plating Medium. Cells (in particular the embryonic population) become elongated and align with neighboring cells within 1–2 days. Terminal differentiation continues, and by the end of day 3, most of the cells have fused. The degree of fusion and the size of mature myotubes are dependent on cell density and the developmental stage of the myogenic progenitors. Progenitors isolated at embryonic stage form small myotubes containing few nuclei, whereas progenitors purified from fetal muscles form large myotubes containing many nuclei. If cultures are protracted beyond day 3, embryonic progenitors will progressively detach from the culture surface and after day 6 only a few elongated cells are adhering to the well. Most fetal progenitors will continue to fuse without detaching until day 6.

**CRITICAL STEP** Use a pipette to remove half of the medium and to slowly add the fresh medium. We usually use a 20–200-μl pipette with filtered sterile tips when we are using 8-well glass chambers slides or 96-well plates. Be careful to not disturb the adherent layer of cells.

## Timing

**Collection of the fluorescent progeny**

Steps 1–2, setting up timed pregnant mice: 5 min per mouse

Steps 3–13, collection of embryos and fetuses: 20 min per pregnant female

Step 14, identification of fluorescent progeny: 20 min per pregnant female.

**Dissection and enzymatic digestion of embryos or fetuses**

Step 15, dissection of embryos and fetuses: 5–10 min per embryo/fetus

Steps 16–25, enzymatic isolation of mononucleated cells: 70–90 min.

**FACS isolation of progenitors and preparation for molecular analysis**

Steps 26–32, cell sorting: 20 min per embryo/fetus

Step 33A, freezing myogenic progenitors for RNA isolation: 20 min

Step 33B, freezing myogenic progenitors for protein purification: 25 min

Step 33C, *in vitro* culturing: 30–35 min to set up.

## Anticipated results

**Figure 8:**
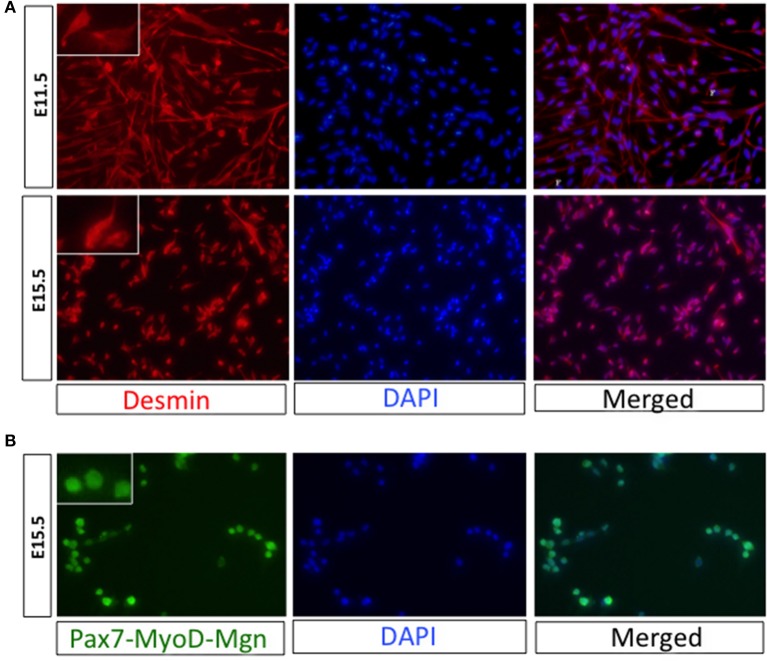
Confirmation of the myogenicity of isolated progenitors. **(A)** Immediately after FACS isolation, cells from *Myf5*^*GFP*−*P*^ embryos (E11.5) or fetuses (E15.5) were plated on collagen-coated wells. Cells were fixed with 4% (wt/vol) paraformaldehyde 12 h after plating and were stained with antibodies recognizing desmin according to standard protocols. **(B)** Cells FACS-purified from the limbs of *Pax7-ICN*^*Cre*/*wt*^*; Z/RED*^*mut*/*wt*^ fetuses (E15.5) were plated on ECM-coated glass chamber slides, fixed as in (A) and stained with a cocktail of antibodies against Pax7, MyoD, and Myogenin (Mgn). A high magnification picture of individual cells is shown in the insets. DAPI was used to stain nuclei.

With this protocol we usually obtain 10,000–100,000 myogenic progenitors per conceptus, depending on the developmental stage, and the mouse strain used for the isolation. For example ~12,000, ~40,000, and ~100,000 cells are obtained respectively from each E11.5 *Myf5*^*GFP*−*P*^ embryo, E15.5 *Myf5*^*GFP*−*P*^ fetus or E11.5 *Pax3*^*GFP*^ embryo. Obviously the number of isolated cells decreases if cells are obtained only from restricted portions of the embryos (for example the limbs). Isolated cells are consistently ≥95% pure when reanalyzed after the sorting for the expression of the reporter gene used for FACS-purification (Supplementary Figure 5), and are highly myogenic (up to 99% when analyzed for myogenic markers 12 h after sorting; Figures 8A,B; Biressi et al., 2007b). The embryonic or fetal lineage of the myogenic progenitors purified with this protocol at different developmental stages can be verified by the quantification of stage-specific markers, such as Arx and Pax3 for embryonic progenitors or Nfix, PKCθ and Itga7 for fetal progenitors (Biressi et al., 2007b, 2008; Messina et al., 2010).

## Notes

Troubleshooting advice can be found in Table 2.

**Table 2:** Troubleshooting table.

**Step**	**Problem**	**Possible reason**	**Solution**
2	Unsuccessful mating Difficult identification of the vaginal plug	Age of the mice or housing conditions are not optimal Plugs of certain strains, such as C57Bl/6 dissolve rapidly	Tips to increase the probability of a successful mating are described in the Jackson Laboratory's website at https://www.jax.org/news-and-insights/jax-blog/2014/september/six-steps-for-setting-up-timed-pregnant-mice Check the plugs early in the morning Use alternative strains such as CD1 or FVB mice when possible
14A	Difficult identification of the fluorescent embryos	Insufficient microscope sensitivity.	Focusing in the bright-field on the myogenic compartment (i.e. limb buds or dermomyotomes) of the embryos before analyzing the fluorescent signal may facilitate the identification Direct comparison with non-fluorescent embryos may also be helpful Use for embryos the same approach described for fetuses (Step 14B)
14B	High auto-fluorescence	Sample is drying out. Skin incompletely removed.	Carefully eliminate skin and bones Process one sample at the time
15	Poor cell yield	Not all myogenic portions are collected	Carefully dissect the tissues by using a stereomicroscope
	Low purity Yeast or bacterial contamination	Incomplete removal of contaminating tissues Unsterile environment or tools	Carefully dissect the tissues by using a stereomicroscope Check at the fluorescence microscope for residual contaminating tissues Dissect by using sterile tools and examination gloves in a sterile hood
18	Yeast or bacterial contamination	Luck of attention to sterile handling techniques	Use standard sterile techniques and open the tubes in a sterile hood
19	Poor cell yield	Incompletely digested tissues are aspirated with the fully disaggregated cells	Do not attempt to aspirate all of the supernatant Aspirate slowly from the surface of the solution Use a 100–1,000 μl pipette to aspirate
22	Poor cell yield and/or purity	Cells are not fully dissociated	Shake vigorously 2–3 times at Step 20 Gently disaggregating with a 5 ml pipette could be an alternative
24	Poor cell yield	The cell suspension is entrapped in the strainer	Change the strainer if it is clogged Carefully recover all liquid from both sides of the strainer
30	Poor cell yield	Presence of dead or apoptotic cells	Optimize the isolation procedure Draw stringent FSC and SSC gates Use live-death dyes or Annexin V conjugates to identify dying or apoptotic cells, respectively
31	Poor purity	Fluorescent cells are not well separated from the background	Optimize the isolation procedure Draw stringent gates to exclude contaminants
32	Sorter clogs/Poor purity	Excessive debris is present Cells have aggregated	Optimize the procedure. Different developmental stages may require for slight modifications in the protocol, in particular at Steps 17–22. Proceed with the sorting immediately after passing the sample trough the strainer Gently vortex or pipette up and down the cell suspension before sorting Filter again through a strainer if necessary Frequently agitate or vortex the sample during sorting
33	Loss of cells	Cells are aspirated	Remove the supernatant slowly from the side of the tube oriented toward the center of the centrifuge to not disturb the pellet
	Yeast or bacterial contamination	Unsterile sorting or culturing conditions	Periodically decontaminate the cell sorter Before sorting the fluidic system should be rinsed with ethanol 70% (vol/vol) Use only sterile cell culture reagents Check for possible contaminations in the filters of the incubator and hood

## Ethics statement

Data reported in this manuscript derive from experiments performed during previous studies. This manuscript was completed without the sacrifice of additional animals.

## Author contributions

All authors contributed to the design of the experiments; GaC, GM, and SB: Conducted and interpreted the experiments; SB, GiC, GM, and EK: Wrote the manuscript.

### Conflict of interest statement

The authors declare that the research was conducted in the absence of any commercial or financial relationships that could be construed as a potential conflict of interest.
