# Supplementary material for: Reporter-Based Isolation of Developmental Myogenic Progenitors
Source: Front Physiol. 2018 Apr 5;9:352. doi: 10.3389/fphys.2018.00352 (PMC5895918; doi:10.3389/fphys.2018.00352)
Supplement: Supplementary file 1 [file Presentation1.pdf]

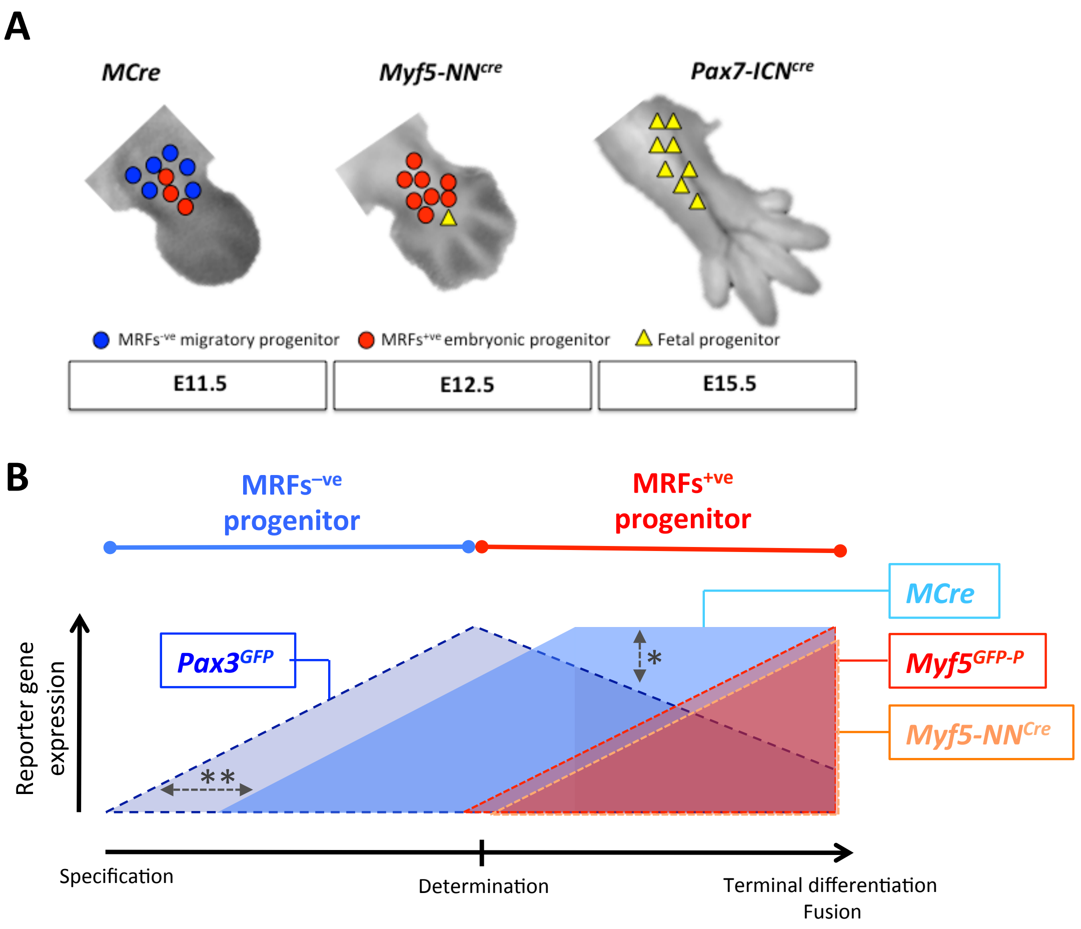


**Supplementary Figure 1. Progenitor isolation from compound mutants relaying on the Cre-LoxP system. (A)** Representation of the numerical proportion and position of distinct subpopulations of myogenic progenitors expressing a fluorescent reporter in limbs of *MCre; Z/RED* and *Myf5-NN*$^{Cre}$*; Z/RED* and *Pax7-ICN*$^{Cre}$*; Z/RED* conceptuses at the indicated stages. Adapted from Boutet et al., 2010, with permission from Elsevier. **(B)** Schematic representation showing the kinetic of the reporter gene expression in the indicated mouse lines during the myogenic lineage progression at embryonic stage. Different steps of the lineage progression are indicated on the x-axis. Note the different pattern of reporter expression in the *Pax3*$^{GFP}$ and *MCre* lines, that is dependent on the constitutive expression of the reporter (*), and on the regulatory sequences used to express Cre in the *MCre* line (**) (Brown et al., 2005).

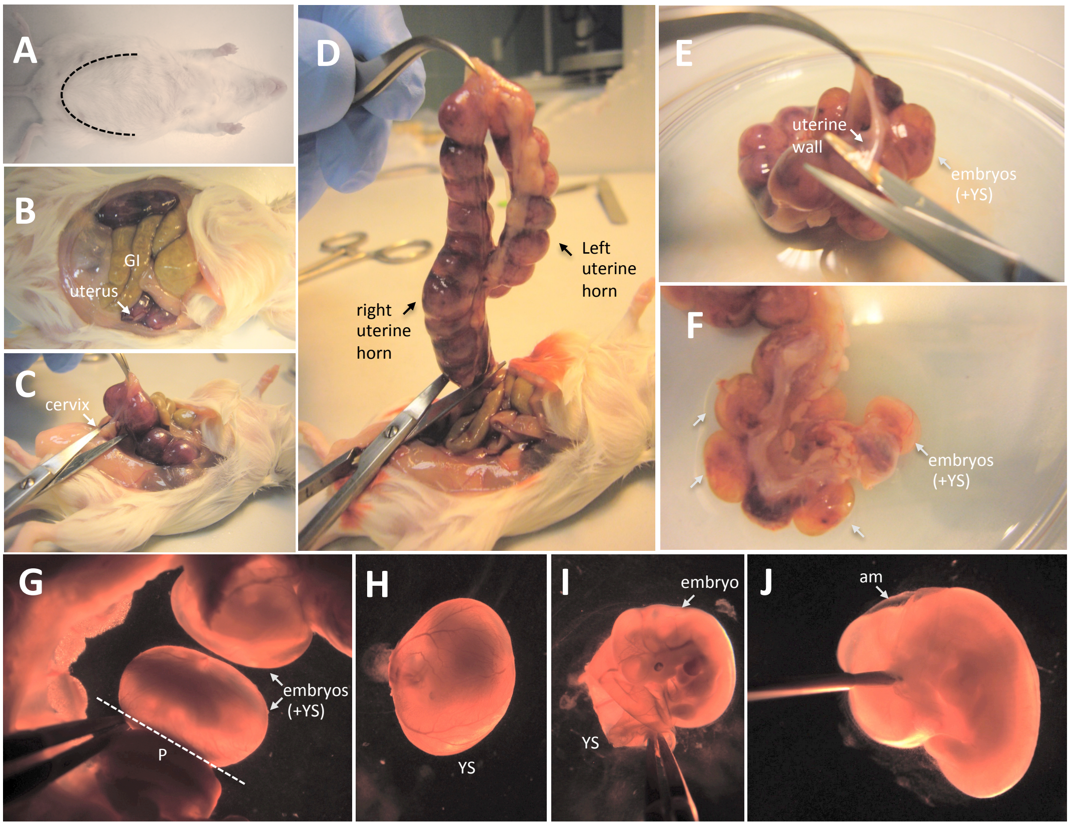


**Supplementary Figure 2. Isolation of a mouse embryo from the uterus.**
**(A)** Incision of the abdomen of a pregnant mouse (dashed line). **(B)** After pulling up the abdominal wall the uterus is visible close to the gastrointestinal tract (GI). **(C)** The cervix is cut with fine scissors. **(D)** The right and left horns are carefully pulled out with forceps. **(E)** While grasping with fine forceps, the uterine horns are cut along their length with scissors. **(F)** Upon removal of the uterine wall mouse conceptuses within their yolk sacs (YS) become clearly visible (arrows). **(G)** A higher magnification of two individual E11.5 mouse conceptuses with the YS and placenta attached and intact. **(H)** An individual E11.5 embryo released from the Reichert's membrane and separated from the placenta with the yolk sac (YS) intact. **(I)** Upon removal of the YS with fine forceps the embryo proper become clearly visible. **(J)** The amnion (am) is visible when the embryo is dissected free from the YS.

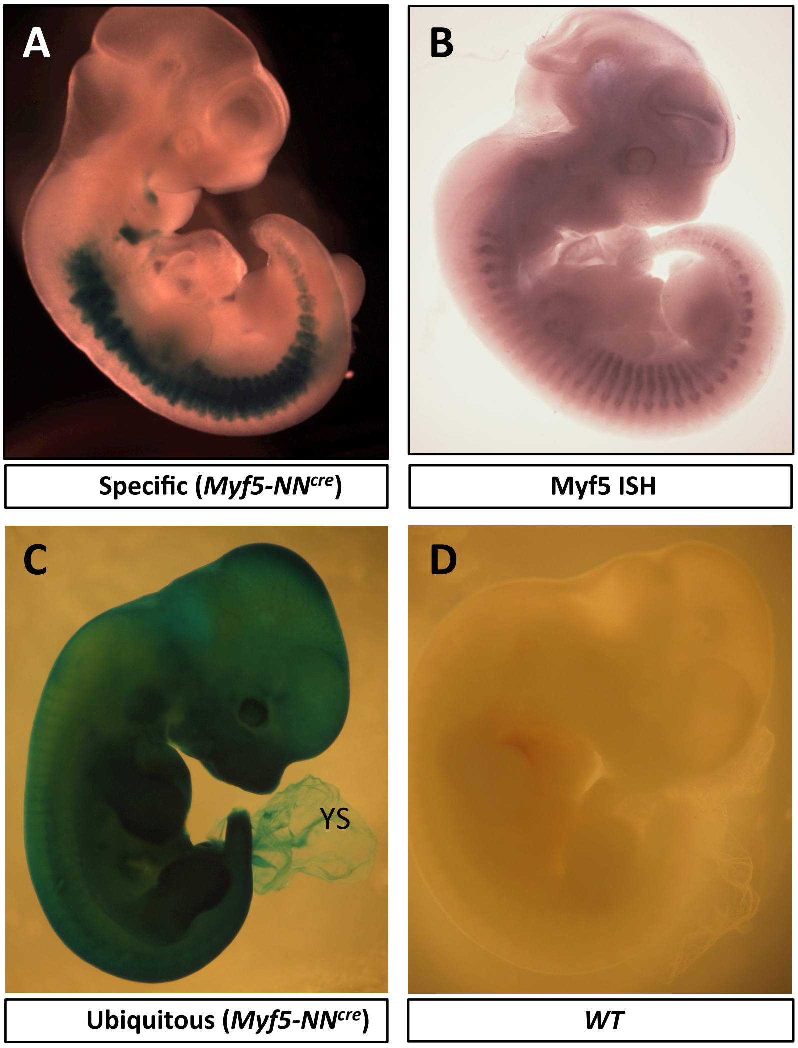


**Supplementary Figure 3. Infidelity of Cre-mediated recombination.** **(A)** Whole-mount X-Gal staining of a *Myf5-NN*$^{Cre}$*; R26R*$^{LacZ/wt}$ E11 embryo presenting a muscle-specific pattern of the reporter gene *LacZ* (Soriano, 1999). **(B)** Whole-mount in situ hybridization (ISH) of an E11 embryo with Myf5-specific probes (Biressi et al., 2013). **(C)** X-Gal staining of a *Myf5-NN*$^{Cre}$*; R26R*$^{LacZ/wt}$ E11.5 embryo showing a ubiquitous pattern of reporter gene expression. Note the widespread staining present in the yolk sac (YS). **(D)** X-Gal staining is absent in *WT* E11.5 embryos (negative control).

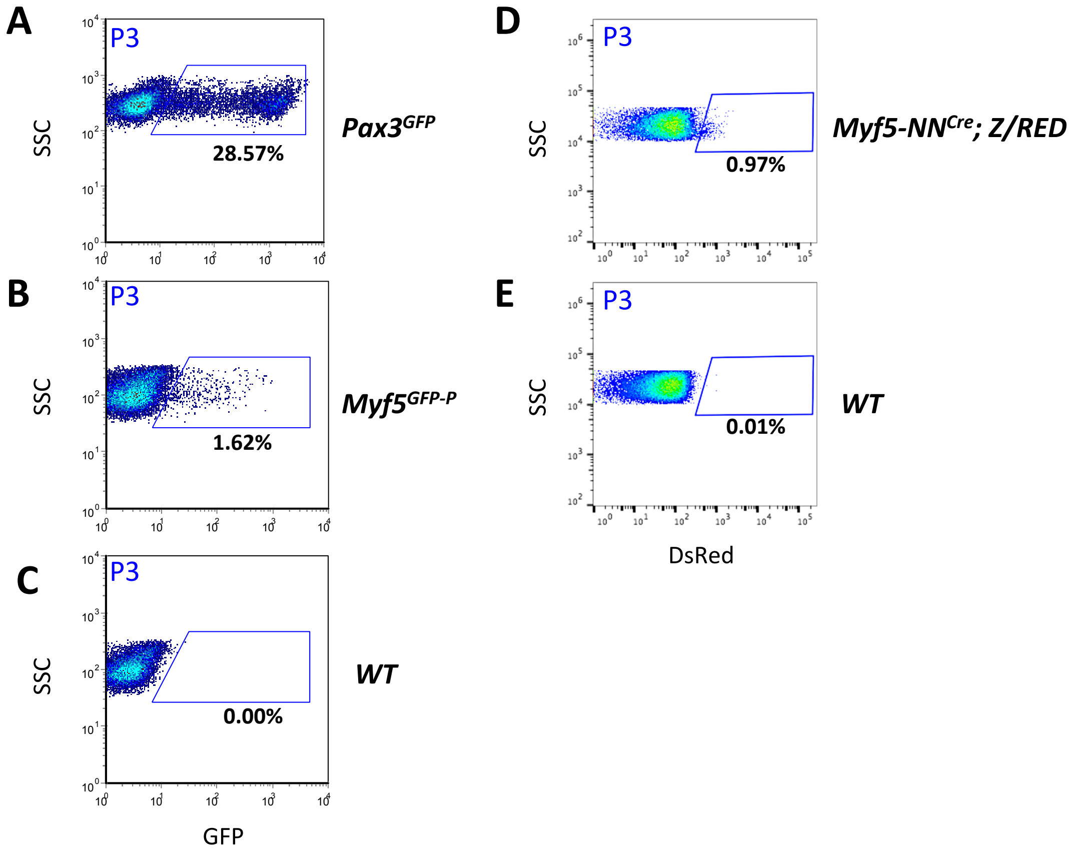


**Supplementary Figure 4. FACS purification of developmental myogenic progenitors. (A)** Two-dimensional dot plot of GFP and SSC of myogenic progenitors obtained after digestion of E11.5 *Pax3*$^{GFP/+}$ embryos.**(B)** Two-dimensional dot plot of GFP and SSC of myogenic progenitors obtained after digestion of E11.5 *Myf5*$^{GFP\text{-}P/+}$ embryos. **(C)** Two-dimensional dot plot of GFP and SSC of cells obtained after digestion of E11.5 *WT* embryos. **(D)** Two-dimensional dot plot of DsRed.T3 and SSC of myogenic progenitors obtained after digestion of the limbs of E14.5 *Myf5-NN*$^{Cre}$*; Z/RED*$^{mut/wt}$ fetuses. **(E)** Two-dimensional dot plot of DsRed.T3 and SSC of cells obtained after digestion of E14.5 *WT* fetuses. Tissue dissociation was performed as described in this protocol. Debris and dead cells are gated out as described for fetal progenitors (Fig. 7). Cells in P3 gate are myogenic progenitors. The quantification of cells in P3 gate obtained in representative experiments is indicated. Modified with permission from Biressi et al., 2008.

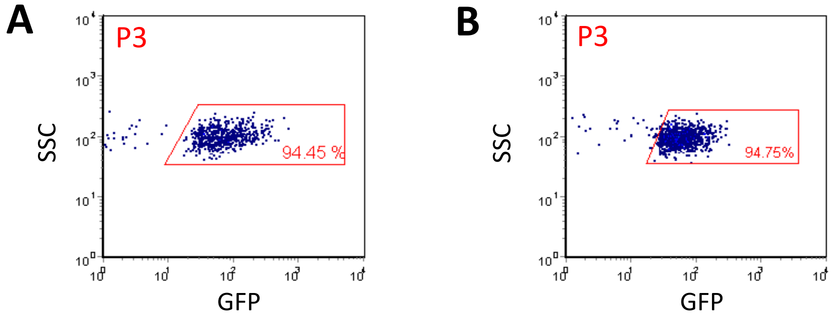


**Supplementary Figure 5. Reanalysis of FACS-purified progenitors.**
**(A)** Two-dimensional dot plot of GFP and SSC of cells reanalyzed after FACS-purification form E11.5 *Myf5*$^{GFP\text{-}P/+}$ embryos. **(B)** Two-dimensional dot plot of GFP and SSC of cells reanalyzed after FACS-purification form E15.5 *Myf5*$^{GFP\text{-}P/+}$ fetuses. Tissue dissociation and FACS-purification were performed as described in this protocol. Debris and dead cells are gated out as described in Fig. 7. Cells in P3 gate are progenitors expressing the reporter gene used for FACS-purification. The quantification of cells in P3 gate obtained in representative experiments is indicated.
